# Improved soil physical properties and cotton root parameters under sub-soiling enhance yield of Cotton-Wheat cropping system

**DOI:** 10.1016/j.dib.2019.103888

**Published:** 2019-03-30

**Authors:** Kulvir Singh, Sudhir Kumar Mishra, Harinder Pal Singh, Angrej Singh, Om Parkash Chaudhary

**Affiliations:** a Punjab Agricultural University, Regional Research Station, Faridkot, Punjab, India; b Soil Science, Punjab Agricultural University, Krishi Vigyan Kendra, Bathinda, Punjab, India; c Soil Chemist Cum Head, Department of Soil Science, Punjab Agricultural University, Ludhiana, Punjab, India

## Abstract

A field experiment has been conducted in Cotton-Wheat cropping system for three cropping cycles, wherein we evaluated a total of five treatments (Control, Sub-soiling at 1.0 m, Sub-soiling at 1.5 m, Cross sub-soiling at 1.0 m and Cross sub-soiling at 1.5 m) in complete randomized block design to find out the effect of sub-soiling on the physical properties of soil and root parameters of cotton in Indian Punjab, where heavy machinery usage in farm operations is causing soil compaction leading to ill effects. Data elucidated that any level of sub-soiling not only improved soil physical properties by reduction in bulk density but also enhanced steady state infiltration rate as compared to control. Data also revealed that root length, fresh root weight plant^−1^ and dry root weight plant^−1^ of cotton exhibited significant differences in sub-soiled plots versus control for initial two years of experimentation but trivial differences existed thereafter. Consequently, both cotton and wheat crop resulted in higher yield owing to above mentioned reasons. The field data set is made publicly available to enable critical or extended analysis.

**Table:** Specifications table

Subject area	*Agriculture*
More specific subject area	*Agronomy, Soil science*
Type of data	*Table*
How data was acquired	*Observations recorded from field experiments*
Data format	*Raw, analyzed, statistical test, etc*
Experimental factors	*Researchers recorded the effect of sub-soiling on soil physical properties, root parameters of cotton, growth and yield of the cotton and wheat crop*
Experimental features	*Five treatments were evaluated and data elucidated that sub-soiling exerted beneficial effect on soil physical properties by reduction in bulk density and improved infiltration rate leading to better growth and yield attributes and consequently improved crop yield*
Data source location	*Faridkot, Punjab, India (300 40′N latitude, 740 44′E longitude, 200 m altitude)*
Data accessibility	*Data is included in this article*

**Table:** 

**Value of the data** • The presented data describe the direct effect of sub-soiling on cotton root growth, productivity and persistence of its residual benefits on Cotton-Wheat cropping system for succeeding crop cycles. This data could be used as reference by researchers for most prevalent cropping systems i.e Rice-Wheat, where puddled field is a pre-requisite before rice transplanting and draw inferences for entire North-western India. • The data describe the effect of sub-soiling on the physical properties of soil and also to find out effective frequency of sub-soiling under site-specific conditions of Indian Punjab. Consequently, this data could provide insights for other researchers working on soil compaction studies for other soil types prevalent in Indo-Gangetic alluvial plains. • The data allows other researchers working on related aspects to extend the statistical analysis and use it in any model evaluation and validation in relation with given weather parameters. This can help in understanding even more serious issues of compaction and remedies in Rice-Wheat cropping cycles.

## 1 Data

The data comprised statistically analyzed raw data on soil physical properties and cotton root parameters at Punjab Agricultural University (PAU), Regional Research Station (RRS), Faridkot, Punjab, (India). Periodical data recorded from the multi-years field experiments conducted at two different sites are described along with weather data for study years.

Table 1, Table 2 shows intra-annual variation in the amount of the rainfall during the study years. Total rainfall was 508.5, 506.1, 385.8 and 376.5 mm for the year 2014, 2015, 2016 and 2017, respectively. This data indicates gradual decrease in the rainfall pattern in recent years and urges to take immediate attention for improving the irrigation facilities/techniques in the scenario of projected climate change [3]. During studied cropping seasons, mean monthly maximum air temperature, minimum air temperature, maximum and minimum relative humidity during studied cropping seasons varied from 27.7 to 40.4 °C, 9.9–27.6 °C, 53–85%, and 22–68%, respectively. Thus, air temperature during the year 2016 remained much higher than the rest years of the study.

**Table 1:** Weather conditions at the experimental site during cropping seasons (2014–2015).

Month	Temperature (°C)				Relative humidity (%)				Total rainfall (mm)		Total evaporation (mm)	
	Maximum		Minimum		Maximum		Minimum					
	2014	2015	2014	2015	2014	2015	2014	2015	2014	2015	2014	2015
April	32.3	34.1	17.7	19.8	60	75	22	49	33.0	45.2	196.8	161.8
May	36.8	40.4	23.4	23.8	60	53	27	27	38.8	2.7	269.1	300.9
June	39.6	37.7	27.3	25.5	63	63	32	38	80.0	134.7	293.2	246.6
July	34.8	34.4	27.6	26.5	79	81	66	65	123.0	162.0	215.0	167.0
August	34.4	34.5	27.0	27.0	83	83	68	67	15.0	118.2	196.8	127.8
September	32.9	34.2	24.3	23.6	78	79	53	52	214.9	41.4	130.9	141.3
October	32.2	32.8	18.3	18.5	83	82	45	41	2.3	1.9	96.6	101.8
November	28.1	27.7	9.9	11.8	85	85	35	36	1.5	0.0	71.8	63.0
Mean/Total	33.9	34.5	21.9	22.1	74	75	44	47	508.5	506.1	1470.2	1310.1

**Table 2:** Weather conditions at the experimental site during cropping seasons (2016–2017).

Month	Temperature (°C)				Relative humidity (%)				Total rainfall (mm)		Total evaporation (mm)	
	Maximum		Minimum		Maximum		Minimum					
	2016	2017	2016	2017	2016	2017	2016	2017	2016	2017	2016	2017
April	36.5	36.9	20.3	25.0	59	60	23	24	2.0	30.3	234.6	232.4
May	40.6	39.5	25.0	26.4	58	54	29	25	28.5	4.6	308.6	286.7
June	39.4	36.8	28.4	28.2	69	69	41	46	72.7	179.1	266.3	237.7
July	35.3	35.5	27.9	27.4	81	80	64	59	91.7	28.6	107.5	187.0
August	33.8	34.7	26.6	23.8	86	82	71	62	190.9	15.6	96.1	150.8
September	34.4	34.0	25.2	17.8	83	86	58	54	0.0	111.1	81.3	102.2
October	34.0	33.9	18.7	10.5	87	88	36	35	0.0	0.0	71.3	94.0
November	28.6	24.5	10.6	11.8	89	94	32	50	0.0	7.2	39.2	37.4
Mean/Total	35.3	34.5	22.8	21.4	77	76	44	44	385.8	376.5	1204.9	1328.2

The data in Table 3, Table 4 on root length, fresh and dry root weight exhibited significant variations among the treatments at both the study sites. At site I, significantly reduced root length of cotton under control i.e 103.2, 98.7 cm was observed during 2014, 2015 respectively as compared to other treatments while trivial differences were observed for year 2016 which indicated that efficacy of sub-soiling is not persistent beyond 2 years (Table 3). Likewise, the root weight data (both fresh as well as dry) for sub-soiled treatments was also significantly higher during 2014 and 2015 whereas for 2016, data exhibited only non-significant differences. Table 4 shows similar data trends for site II during 2015.

**Table 3:** Root parameters of cotton under different sub soiling treatments at Site I (2014).

Treatments	2014			2015			2016		
	RL	FR	DR	RL	FR	DR	RL	FR	DR
Control (No sub-soiling)	103.2a	46.2a	14.1a	98.7a	35.9a	12.3a	102.3a	45.8a	13.9a
Sub-soiling at 1.0 m	146.5b	64.7b	21.8b	123.5b	52.9b	16.4b	102.8a	51.6a	14.5a
Sub-soiling at 1.5 m	144.5b	65.7b	22.9b	124.5b	55.1b	17.1b	100.9a	44.9a	13.8a
Cross sub-soiling at 1.0 m	147.2b	66.0b	24.2b	125.4b	54.9b	17.5b	98.6a	47.7a	14.0a
Cross sub-soiling at 1.5 m	150.2b	67.2b	22.4b	123.6b	55.6b	18.0b	105.6a	45.6a	14.1a

Note: 1. RL: Root length (cm); FR: Fresh root weight plant^−1^ (g); DR: Dry root weight plant^−1^ (g).

2. Sub-soiling has been given only once during 2014 prior to sowing of cotton.

3. Means in the same column followed by different lowercase letters differ significantly from each other based on LSD (0.05).

**Table 4:** Root parameters of cotton under different sub soiling treatments at Site II (2015).

Treatments	2015			2016			2017		
	RL	FR	DR	RL	FR	DR	RL	FR	DR
Control (No sub-soiling)	92.8a	37.4a	13.3a	102.5a	38.5a	12.6a	106.9a	39.6a	14.4a
Sub-soiling at 1.0 m	135.7b	58.3b	22.0b	130.9b	53.4b	19.5b	108.7a	40.0a	14.9a
Sub-soiling at 1.5 m	134.8b	56.2b	21.2b	132.5b	55.9b	19.8b	102.6a	37.6a	13.8a
Cross sub-soiling at 1.0 m	139.2b	64.2b	25.4b	137.6b	56.2b	20.0b	105.9a	38.3a	13.6a
Cross sub-soiling at 1.5 m	140.9b	62.1b	23.3b	134.7b	54.8b	19.5b	107.4a	39.7a	14.0a

Note: 1. RL: Root length (cm); FR: Fresh root weight plant^−1^ (g); DR: Dry root weight plant^−1^ (g).

2. Sub-soiling has been given only once during 2015 prior to sowing of cotton.

3. Means in the same column followed by different lowercase letters differ significantly from each other based on LSD (0.05).

Table 5 shows that at site I during 2014 cropping season, the infiltration rate at the start of experiment was significantly higher under cross sub-soiling, while it was statistically least under control. However, a gradual decline in infiltration rate with each successive cropping season was recorded in subsequent year's i.e 2015 and 2016. Data on control plots for site I, revealed highest values for bulk density. Data on bulk density at site I show that most of the variation existed in 15–30 and 30–60 cm soil depth while at 0–15 cm, non-significant differences prevailed. Bulk density data exhibited non-significant differences for stage III, which elucidated that persistence of sub-soiling effect existed no longer than two seasons. Data in Table 6 shows a similar trend for bulk density and infiltration rate for site II as that of site I. Sub-soiling improved cane productivity [5] and wheat yield [7] over the respective control by increased infiltration rate. Higher values (≥1.67 g cm^−3^) have been observed for control under 30–60 cm soil depth as compared to rest of profiles and bulk density higher than 1.6 g cm^−3^ hampers root growth [4], [6].

**Table 5:** Steady state infiltration rate and soil bulk density under different sub soiling treatments at Site I.

Treatments	Steady state infiltration rate (mm hr^−1^)			Bulk density of soil (g cm^−3^)								
				Stage I			Stage II			Stage III		
	Stage I	Stage II	Stage III	0–15 cm	15–30 cm	30–60 cm	0–15 cm	15–30 cm	30–60 cm	0–15 cm	15–30 cm	30–60 cm
Control (No sub-soiling)	2.2a	1.9a	2.0a	1.51a	1.58a	1.67a	1.50a	1.59a	1.69a	1.52a	1.60a	1.69a
Sub-soiling at 1.0 m	3.0b	2.5ab	2.2a	1.45a	1.46b	1.49b	1.46a	1.48b	1.53b	1.50a	1.52a	1.60a
Sub-soiling at 1.5 m	3.3b	2.8bc	2.1a	1.44a	1.48bc	1.51b	1.48a	1.52bc	1.55b	1.51a	1.56a	1.59a
Cross sub-soiling at 1.0 m	4.1c	3.2c	2.2a	1.40a	1.50bc	1.49b	1.49a	1.53c	1.54b	1.50a	1.55a	1.58a
Cross sub-soiling at 1.5 m	4.3c	3.0c	2.1a	1.41a	1.52c	1.51b	1.47a	1.54c	1.54b	1.51a	1.57a	1.59a

Note: Stage I: June, 14 (at start of experiment); Stage II: Oct, 15 (mid stage); Stage III: Oct, 16 (at end of experiment).

Means in the same column followed by different lowercase letters differ significantly from each other based on LSD (0.05).

**Table 6:** Steady state infiltration rate and soil bulk density under different sub soiling treatments at Site II.

Treatments	Steady state infiltration rate (mm hr^−1^)			Bulk density of soil (g cm^−3^)								
				Stage I			Stage II			Stage III		
	Stage I	Stage II	Stage III	0–15 cm	15–30 cm	30–60 cm	0–15 cm	15–30 cm	30–60 cm	0–15 cm	15–30 cm	30–60 cm
Control (No sub-soiling)	1.9a	1.8a	1.8a	1.55a	1.57a	1.69a	1.56a	1.59a	1.71a	1.60a	1.64a	1.71a
Sub-soiling at 1.0 m	2.5b	2.4b	1.8a	1.53a	1.56a	1.60b	1.54a	1.57a	1.66b	1.57a	1.60a	1.67a
Sub-soiling at 1.5 m	2.4ab	2.2b	1.9a	1.51a	1.56a	1.59b	1.55a	1.58a	1.68a	1.58a	1.60a	1.67a
Cross sub-soiling at 1.0 m	2.7b	2.4b	1.9a	1.43a	1.51b	1.57b	1.46a	1.52b	1.62c	1.52a	1.59a	1.65a
Cross sub-soiling at 1.5 m	2.6b	2.3b	2.0a	1.47a	1.49b	1.58b	1.49a	1.53ab	1.64bc	1.53a	1.59a	1.65a

Note: Stage I: June, 15 (at start of experiment); Stage II: Oct, 16 (mid stage); Stage III: Oct, 17 (at end of experiment).

Means in the same column followed by different lowercase letters differ significantly from each other based on LSD (0.05).

## 2 Experimental design, materials and methods

Field experiment was conducted for three consecutive crop cycles (2014–2017) at two different sites at Regional Research Station, Faridkot, India (latitude 30° 40′N, longitude 74° 44′E and altitude 200 m above mean sea level). At site I, three Cotton-Wheat cropping cycles were studied from 2014 to 2017. At site II, three cotton crops and two wheat crops were evaluated starting from 2015 to 2017. At both sites, experiment was laid out in completely randomized block design having four replications. There were five treatments i.e. Control, Sub-soiling at 1.0 m, Sub-soiling at 1.5 m, Cross sub-soiling at 1.0 m and Cross sub-soiling at 1.5 m. Sub-soiling at both the sites was performed before sowing of cotton crop during initial year only by using a tractor drawn sub-soiler (*Chiseler*) to a depth of 45–50 cm at the time of field preparation just before sowing. Each treatment plot (42.5 m^2^) accommodated 84 cotton plants in a planting geometry of 67.5 × 75 cm. *Bt* cotton cultivar NCS855 BGII was planted during 2014 and 2015, while RCH650 BGII was grown during 2016 and 2017. Wheat Cv. HD 2967 was studied during 2014 and 2015 while during 2016 Cv. PBW 725 was grown. Bulk density of soil was measured using core method [1] and *in situ* steady state infiltration rate were recorded using double ring infiltrometer in field [2].
